# Spatial Variability of External Egg Quality in Vertical Naturally Ventilated Caged Aviaries

**DOI:** 10.3390/ani13040750

**Published:** 2023-02-19

**Authors:** Letícia Cibele da Silva Ramos Freitas, Ilda de Fátima Ferreira Tinôco, Richard Stephen Gates, Tatiany Carvalho dos Santos, Rafaella Resende Andrade, Matteo Barbari, Gianluca Bambi

**Affiliations:** 1 Department of Agricultural Engineering, Federal University of Viçosa (UFV), Viçosa 36570-900, MG, Brazil; 2 Egg Industry Center, Departments of Agricultural and Biosystems Engineering, and Animal Science, Iowa State University, Ames, IA 50011, USA; 3 Department of Agriculture, Food, Environment and Forestry, University of Firenze, 50145 Firenze, Italy

**Keywords:** eggshell quality, laying hens, spatial distribution, thermal conditions

## Abstract

**Simple Summary:**

The external quality of eggs is an essential factor to consider in the poultry production sector since economic losses are directly related. This study evaluated the spatial variability of external egg quality in naturally ventilated vertical caged aviaries, with variability in quality hypothesized to be caused by hen age differences and by variability in thermal and light conditions within the aviary. Both winter and summer conditions in a tropical region were examined. The spatial variability of external egg quality was found to be greater in summer than in winter, regardless of hen age. Egg weight and shell quality values were lower in the upper level of cages located in the center of the aviary. The light intensity in the aviary presented tremendous spatial variability, but its effect on external egg quality was not significant. Egg external quality spatial distribution determination is shown to be a useful indicator of economic loss and an aid in hen nutritional and thermal management, as well as in egg collection and classification, by deploying a differentiated management of collection in the area where hens produce eggs with lower external egg quality.

**Abstract:**

External egg quality is an essential parameter of egg production as it relates directly to economic losses. This study evaluated the spatial variability of external egg quality in five naturally ventilated caged vertical aviaries. Differences caused by bird age and thermal and luminous variability within aviaries during winter and summer were analyzed. Data on aviary air temperature, relative humidity, light intensity, and external egg quality were collected at evenly distributed points along the aviary length within three levels of cages. The experimental design was completely randomized in a factorial scheme. In the summer, the highest air temperature and lowest relative humidity were found in central cages, mainly in upper center cages; hens produced eggs with a lower weight and shape index in this area. Similar results were obtained in the winter. In the summer, eggs with lower shell weight and thickness were also produced by hens housed in the central cages, but in the winter, the opposite result was obtained. This study of the spatial variability of external egg quality proved efficient in detecting areas within an aviary with poor quality eggs; improvements to design and management in these areas could help management improve production efficiency and contribute to a sustainable egg supply.

## 1. Introduction

Egg quality is characterized by a set of external and internal egg parameters that affect the production process, economics, and profitability. The external quality of eggs is an essential factor to consider in the egg production sector since economic losses are directly related. Egg weight and shape index are two parameters used in the commercial classification of eggs and are directly related to the propensity for breakage. The parameters frequently analyzed in determining external egg quality are egg weight, shape index, specific gravity, shell weight, shell thickness, and shell mass percentage of the egg [1].

The egg shape index is defined as the relationship between the egg’s width and length and positively correlates with egg weight [2,3]. Egg weight is a parameter used in commercial egg classification, and the shape index is used to detect unusually shaped eggs, such as long or round eggs. Oval eggs do not fit snugly into egg cartons and are therefore more likely to be broken during loading and transport than normal-shaped eggs [2].

The eggshell acts as a protective barrier against contamination by microorganisms and controls the exchange of gases through the pores during incubation [4]. The external egg quality parameters related to the protection function are shell weight, shell thickness, and shell percentage. The analysis of these parameters is important to keep the eggs intact and free of contaminants for consumption.

Egg specific gravity is an indirect parameter commonly used to analyze eggshell strength. Specific gravity is related to the shell percentage and shell thickness of the egg. Shell percentage is the ratio of egg shell weight to egg weight. Therefore, the higher the percentage of shell and thickness, the higher the specific gravity, and thus the more resistant the eggshell [5].

Bird age, as well as variations in thermal condition and light intensity within an aviary, are factors that have been seen to influence external egg quality. As hens age, egg weight increases and shell weight, percentage of shell, and shell thickness [6,7] all decrease. Exposure to air temperatures above the thermal comfort zone [8,9,10] negatively influences the external egg’s quality [11,12,13], and its effect will be greater with higher levels of air relative humidity in the air [14]. Light intensity negatively affects the external quality of eggs [15,16,17,18,19]. A lux intensity of 10 at the hen’s head is considered necessary to produce quality eggs in open aviaries [20,21].

The study of the simultaneous effects of thermal and light conditions in the interior of the aviary and the age of the birds can provide relevant information about the region of the aviary that produces lower-quality eggs. Therefore, the present study aimed to evaluate the spatial variability of external egg quality in naturally ventilated vertical aviaries as affected by the age difference between the birds and by the thermal and light conditions of the interior of the aviary in winter and tropical summer.

## 2. Materials and Methods

### 2.1. Characteristics of Facilities

The experiment was carried out in July 2016 (winter) and January 2017 (summer) in five aviaries with a vertical cage system and natural ventilation, located on a farm in the southern region of the state of Minas Gerais, Brazil, at geographic coordinates 22°17′45″ S and 44°56′05″ W at an average elevation of 892 m. The aviaries were 134.0 × 12.5 m (length × width), 5.0 m high, 1.2 m open ridge (covered); the aviaries were constructed with metallic frames and an uninsulated galvanized steel roof.

The facility housed four batteries of vertical cages, each with six floors. Each cage had dimensions of 0.6 × 0.5 × 0.4 m (width × depth × height), housing ten birds per cage with an average density of 300 cm^2^.bird^−1^ (high density). Each aviary on the farm could accommodate up to 100,000 laying hens.

**Figure 1:**
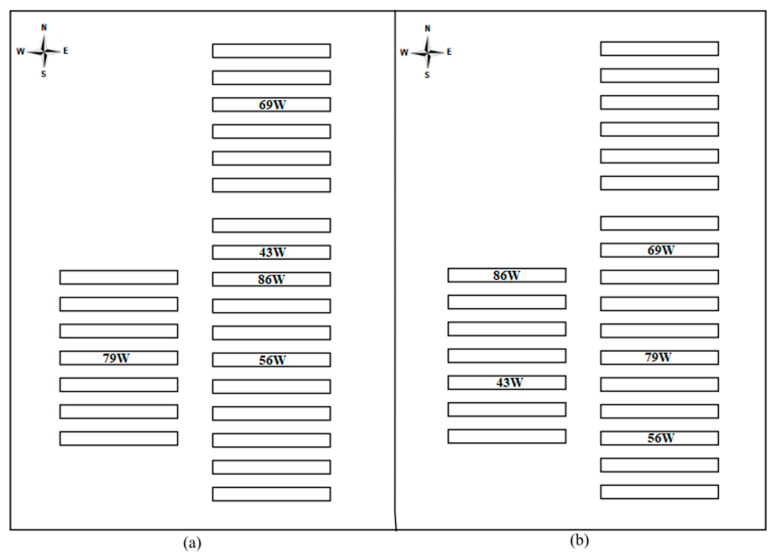
Aviary locations layout with hens at 43 weeks (43W), 56 weeks (56W), 69 weeks (69W), 79 weeks (79W), and 86 weeks (86W) of age (**a**) in winter and (**b**) summer.

The aviaries utilized in this study housed Hy-Line W-36 laying hens at different ages (43, 56, 69, 79, and 86 weeks of age), both in winter and summer. Hens’ ages were determined according to the farm’s production schedule, and they were selected to allow for studying birds of the same age in both winter and summer. Figure 1 displays the aviaries’ location within the farm complex. As can be seen, over the two seasons, there were eight independent aviaries included in the study, five for each season, with winter aviary 43W being the same as that labeled summer aviary 69W and winter aviary 56W being labeled as summer aviary 79W. This was done to ensure that five unique hen ages could be consistently compared between the two seasons.

The farm’s light program was 16 h of light (a combination of natural and artificial) and 8 h darkness, as recommended in the Hy-Line W-36 Management Manual [22]. Feed was supplied through automatic feeders and water through nipple drinkers, both ad libitum. The collection and transport of eggs and the collection and removal of feces were done with motorized egg and manure belts, respectively.

### 2.2. Thermal Conditions and Light Intensity

**Figure 2:**
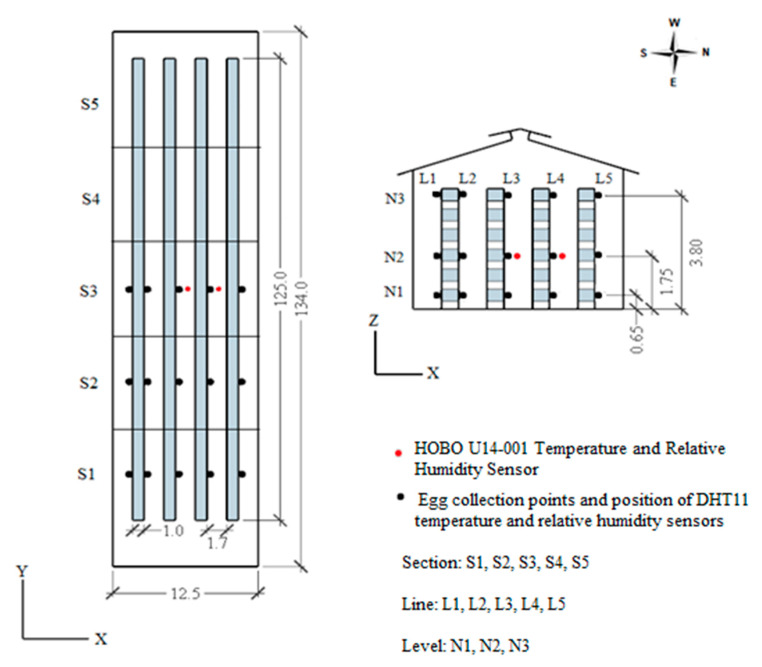
Spatial location of egg collection points and temperature (°C) and relative air humidity (%) sensors. Dimensions in meters.

In the five aviaries evaluated during each season (Figure 1), temperature and relative humidity sensors (HOBO U14-001, Onset Computer Corp., Bourne, MA, USA) were installed in lines L2 and L3 of level N2 of section S3 (Figure 2) for six winter days and six summer days. Air temperature and relative humidity were measured from 6:00 a.m. to 6:00 p.m. every five minutes to assess the thermal environmental variability between the aviaries.

One aviary was chosen to assess the spatial distribution of the thermal condition during the evaluation of external egg quality variability, as it was determined from the prior evaluation that there were no significant temperature differences between the five aviaries and minimal differences in relative humidity. For this single aviary, temperature and relative humidity sensors (DHT-11, Digi-Key Electronics, Thief River Falls, MN, USA) were installed at 45 points distributed uniformly along the longitudinal (y) and transverse (x) directions (Figure 2). These data were collected every five minutes for four consecutive days in both winter and summer.

The sensors were distributed in these three aviary sections because these sections displayed the greatest variability in summer air temperature in earlier work by Freitas et al. [23]. At the 45 points where the temperature and relative humidity sensors were installed (Figure 2), light intensity values were also measured daily using a lux meter (MLM-1011, MINIPA, São Paulo, Brazil) at 9:00 a.m., 12:00 p.m., and 3:00 p.m. for three days.

### 2.3. External Egg Quality

The spatial variability of the external egg quality inside the aviaries was evaluated in both winter and summer, simultaneously in five aviaries with hens at 43, 56, 69, 79, and 86 weeks of age. Eggs were collected from 45 points, distributed vertically and longitudinally in the aviaries, for four days each of winter and summer. Each collection point represents a set of coordinates (x, y, z), with the direction of the *x*-axis being the lines (L1, L2, L3, L4, L5), the *y*-axis direction being the sections (S1, S2, S3), and the *z*-axis direction being the levels (N1, N2, N3), as shown in Figure 2. Levels N1, N2, and N3 corresponded to the first, third, and sixth battery cages, respectively.

Installation of barriers between the cages and the egg conveyor belt was done the day before egg collection. These barriers prevented the passage of eggs to the conveyor belt, thus ensuring that the collected eggs were from the cages under study.

Egg collection was performed between 7 and 8 a.m., and afterword, the barriers were removed. This process was repeated for four days of collection, and the eggs were analyzed after collection. A 24-h delay interval between temperature and relative humidity collection and egg collection was enforced to better relate how thermal history impacted an egg’s external quality metrics. In total, 45 eggs/day were analyzed in each aviary, for a total of 900 eggs per season.

The external quality parameters evaluated were egg weight (g), egg shape index (%), shell weight (g), shell percentage (%), shell thickness (mm), and specific gravity (g·mL^−1^). Egg weight was obtained by weighing the eggs on a digital scale with a precision of 0.01 g. The egg shape index was determined by the ratio between the smallest diameter of the egg (W, the equatorial width) and the largest diameter of the egg (L, the length), each measured with a caliper.

Specific gravity was determined by immersing eggs in saline solutions (NaCl) with densities between 1050 and 1100, with minimum intervals of 0.005 as measured by a densimeter per Hamilton [5]. The eggs, when floating, were classified according to their specific gravity.

The shells were washed under running tap water to remove albumen residuals, dried at room temperature for 24 h, and individually weighed on a 0.01-g precision digital scale. Shell percentage was calculated by dividing shell weight by egg weight. The shell thickness was taken to be the average of values measured at three points along the shell’s equatorial region using an external micrometer after drying for 24 h.

All procedures adopted in this study followed the ethical principles of animal experimentation approved by the Commission on Ethics in the Use of Animals (CEUA) of the Federal University of Viçosa (UFV), Minas Gerais, Brazil, with registration protocol number 46/2016.

### 2.4. Statistical Analysis

Assessment of spatial variability of external egg quality was done using a completely randomized design (CRD). Effects of spatial variability were evaluated using a simple analysis of variance, and means separation was performed using a Tukey test at 5% probability. The R Software, version 3.4.1, was used for statistical analysis, and the Surfer Software, version 15.0, for creating the graphs.

To assess the similarity of the thermal conditions of the aviaries in winter and summer, an analysis of variance was conducted with the daily values of temperature and relative humidity, measured in the five aviaries (treatments) for six days (replicates). From this analysis, which showed no significant temperature effects and limited differences in relative humidity, one representative aviary was chosen to determine the spatial distribution of the thermal conditions in its interior during the period when external egg quality measurements were taken. An analysis of the daily air temperature and relative humidity measured during four sampling days (replications) in winter and summer was performed as a 3 × 3 × 5 factorial scheme. The factors corresponded to the level (N1, N2, N3), section (S1, S2, S3), and line (L1, L2, L3, L4, L5).

In this same aviary, the spatial distribution of light intensity was also assessed, in a similar 3 × 3 × 5 factorial scheme, with mean values of light intensity measured at 9:00 a.m., 12:00 p.m., and 3:00 p.m. on the three days of collection (replications).

An independent 2 × 5 factorial analysis, with four replications, was performed to assess the effects of season (winter vs. summer) and hen age (43, 56, 69, 79, and 86 weeks) on the external quality of eggs. From the result of this analysis, the spatial variability of the external quality of eggs inside the aviaries in winter and summer at different hen ages was determined.

The spatial variability of the external quality of eggs inside the aviaries, in both winter and summer, was determined using a 3 × 3 × 5 factorial scheme with four replications (collection days) of the parameters egg weight (g), index of egg shape (%), specific gravity (mg·L^−1^), shell weight (g), shell percentage (%), and shell thickness (mm). The factors corresponded to the level (N1, N2, N3), section (S1, S2, S3), and line (L1, L2, L3, L4, L5) as shown in Figure 2. Each collection point corresponded to a cage (an experimental unit), from which eggs were taken for external quality analysis.

## 3. Results and Discussion

In winter, the aviaries’ average interior daily temperature and relative humidity were not significantly different, nor were the summertime average daily temperatures (*p* > 0.05). However, the mean daily summer relative air humidity was different (*p* < 0.05), as Table 1 points out, although with a range of only 4% in mean daily values between Aviary 43W and Aviaries 69W and 86W.

The significant but small magnitude variation in relative humidity among aviaries during the summer may be due to the predominance of rain and northwesterly winds, which directed humid air into the interior of the aviary, mainly the leeward region (Figure 1b). The winds were generally from a northerly direction in winter, falling directly on the aviaries with exposed sides (Figure 1a). These aviaries prevented the wind from flowing to the adjacent aviaries, generating homogeneity in the interior relative humidity.

Due to the interference of prevailing winds, the geographical position of the aviaries, and the statistical difference highlighted in Table 1, the 43W aviary was chosen to assess the spatial variability of the thermal conditions during sampling for external egg quality (Figure 2). As a result, the average values of temperature and relative humidity in winter and summer varied according to levels (N) and lines (L), and the N × L interaction was significant (*p* < 0.001), as shown in Table 2.

**Figure 3:**
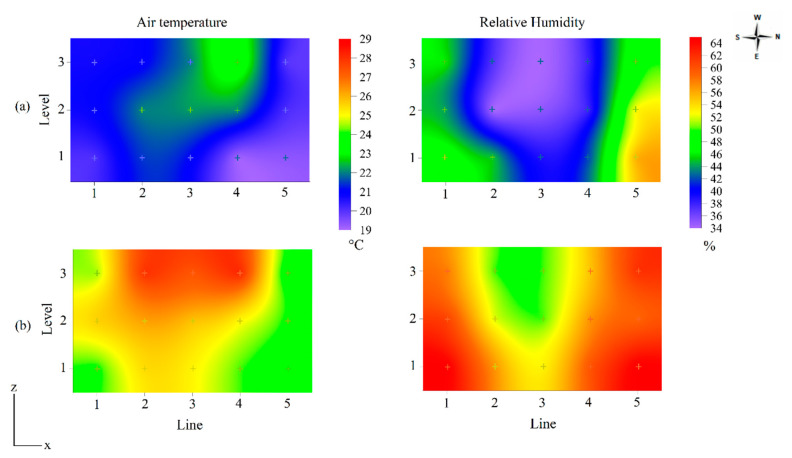
Spatial distribution of air temperature and relative humidity inside the aviary with 43-week-old laying hens (43W), in winter (**a**) and summer (**b**).

The interaction between levels and lines was visualized through the spatial distribution of temperature and relative humidity values obtained inside the 43W house in cross-section (lines × levels), as shown in Figure 3.

During winter, inside the house, the level N1 air temperature varied between 19 and 21 °C, with the highest values in lines L2 and L3. At level N2, there was an increase in temperature in the center lines of the house (L2, L3, and L4), with values around 22 °C. At level N3, the heat concentrated around the L4 line, with a mean of 23 °C.

Interior relative humidity in winter was low and varied between 34% and 56%. The dry air concentrated in the center of the aviary with values around 35%, where the air temperature was greatest. The sides of the aviary, being open and exposed to the external environment, presented higher relative humidity values, between 44% and 56%.

During summer, the lowest air temperature values were measured in lines L1 and L5 at all levels. The hot air was concentrated in the center lines of the aviary, suggesting a need for greater ventilation, with the highest temperature values, between 27% and 28 °C, observed at level N3. The relative humidity of the air was around 50% to 65%; as in winter, the dry air was concentrated in the center of the aviary, ranging between 50% and 57%.

In mechanically ventilated caged aviaries, Zheng et al. [24] found interior barn temperatures ranging from 26.5 ± 1.1 to 28.1 ± 1.1 °C during winter periods with outside temperatures of 13.0 ± 4.8 °C; and 29.1 ± 1.1 to 29.6 ± 1.2 during summer periods with outside temperatures of 26.0 ± 3.1°C. The naturally ventilated aviaries in this study were generally more uniform in temperature than the US facility studied by Zheng et al. [24] and also in the three different systems (conventional cage, natural mating colony, cage-free aviary) studied by Li et al. [25]. However, the more uniform and benign tropical climate in the present study also affected these differences in interior environment variability [26].

Under heat stress conditions, there is a reduction in the concentrations of vitamins, minerals, and insulin available for metabolism and an increase in mineral excretion [13], which causes laying hens to produce poor-quality eggs [1].

The variability of light intensity in the 43W house was substantial in both winter and summer. There was a significant effect of the lines, levels, and the interaction between them (*p* < 0.001), as shown in Table 3.

The lower light intensity in the center (L2, L3, and L4) was generated predominantly by artificial lighting, whereas the higher intensity on the sides (L1, L5) of the house, both in winter and summer, was from direct exposure to the sky. In winter, only the L3-level N1 line presented an average light intensity value close to 10 lux, as recommended by Cotta [20] and Jácome et al. [21].

The high values of light intensity in winter on the L1 line can be explained by the direct incidence of the sun’s rays due to the latitude of the farm (22°17′45″ S and 44°56′05″ W). In summer, there is no direct incidence of radiation, and the light intensity values in the L1 line were below those measured in winter.

Egg weight was significantly affected by the factors “season of the year″ and “age of hens″ (*p* < 0.001) and their interaction (*p* < 0.01). On the other hand, egg shape index and specific gravity were only affected by the season of the year factor (*p* < 0.05). Regarding the quality of the shell, there was a significant effect of the seasonal factor on shell weight (*p* < 0.001) and shell thickness (*p* < 0.05), and, for the age factor, on the shell percentage (*p* < 0.01). Table 4 lists the significant effects of the factors and the interactions between them on the egg quality parameters. The external egg quality parameters that were not affected by age hens and season of the year (*p* > 0.05) are presented in Table 4 and their means and standard deviation were followed by “ns”.

Older birds produced heavier eggs, as expected. With increasing age, hens produce larger follicles [27] and have a greater capacity to transfer lipids to the yolk [28], stimulating heavier egg production. This relationship between hens’ age and egg weight is well documented (e.g., Dirkmen et al. [29], Samiullah et al. [7], and Onbaşilar et al. [6]).

The influence of thermal conditions inside the aviaries was more accentuated for laying hens aged 69, 79, and 86 weeks. At these ages, there was a significant decrease in egg weight in the summer compared to the winter. Birds in environments with high temperatures reduce food intake, leading to metabolic changes [14] and, consequently, reduced egg quality.

Seasonal and location effects in the aviary had little influence on specific gravity. The average value of specific gravity was 1.086 g·mL^−1^ and 1.090 g·mL^−1^ in winter and summer, respectively. These values are higher than those reported by Torki et al. [13] for laying hens subjected to cold (17 °C) and heat stress (32 °C).

The shape index is directly affected by egg weight and indirectly by winter and summer thermal conditions. At cooler temperatures, hens tend to ingest and metabolize food better, producing heavier eggs. These eggs concentrate albumen (the dense part of the egg) in the central equatorial region, which gives a greater shape index [2].

Shell thickness values varied between seasons, with thinner shells in the summer than the winter. Pulmonary hyperventilation occurs due to hen’s increased respiratory rate when exposed to high-temperature environments. Pulmonary hyperventilation causes a reduction in the blood HCO_3_ and CO_2_ levels, which increases the blood pH and causes respiratory alkalosis. Consequently, there is a reduction in the synthesis of calcium carbonate, which is necessary for the formation of the shell [20,30], which makes them thinner and lighter in weight.

Eggs from hens aged 56 weeks had a higher shell percentage than eggs from hens aged 86 weeks. Castro et al. [31] subjected laying hens to thermal comfort (21 °C) and heat stress (32 °C) conditions during the period of 19 to 45 weeks of age and observed that heat stress conditions did not affect the shell percentage; Hu et al. [1] found no difference in egg weight, shell weight or thickness, or shell percentage for 16–32-week-old hens subjected to a short-term heat stress event. In this study, hens exposed to heat stress for 45 weeks produced eggs with a shell percentage of 9.13%, corroborating the present work because no statistical differences in shell percentage values were found between winter and summer.

With advancing age, there is less intestinal calcium absorption and a higher removal of calcium from the bones. As a consequence, the calcium content available for the formation of the shell decreases, reducing its weight [20]. Therefore, older birds tend to produce heavier eggs with lighter shells, resulting in a low shell percentage, as verified in the present work and by Dirkmen et al. [29] and Samiullah et al. [7].

The variability of thermal conditions in the aviaries in winter and summer affected external egg quality. The spatial distribution of egg quality was studied in aviaries housing laying hens aged 43 and 86 weeks (Aviary 43W and Aviary 86W). These two aviaries were chosen to analyze the spatial distribution due to the significant bird age effect on the parameters of egg weight and shell percentage, as observed in Table 4.

In the 43W aviary (43 weeks old hens), there was a significant difference between the weight of eggs obtained from the lines (*p* < 0.1) and levels (*p* < 0.1) in winter and between levels (*p* < 0.001) in summer. In the 86W house, the line factors (*p* < 0.01) and level (*p* < 0.1) and the line × level interaction (*p* < 0.01) were significant in winter, as well as the line (*p* < 0.01) and level (*p* < 0.05) and the line × level interaction (*p* < 0.05) in summer. Table 5 displays the effect of the level (N) and line (L) factors on the mean egg weight (g) value obtained in 43W aviaries in winter and summer and the effect of the interaction N × L average egg weight (g) obtained in 86W aviaries in winter and summer. There was no significant effect (*p* > 0.05) of the line factor (L) on the average value of egg weight (g) obtained in 43W aviaries in the summer, therefore, in Table 5 the means and standard deviation were followed by “ns”.

**Figure 4:**
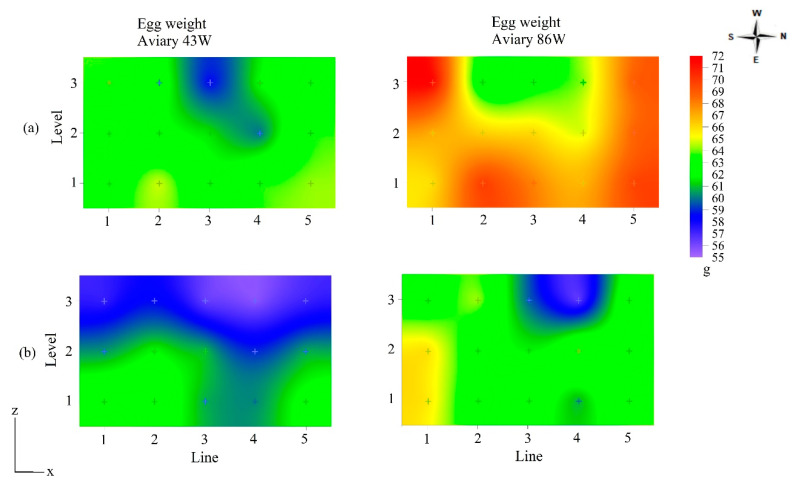
Spatial distribution of egg weight in two aviaries with 43 and 86 week-old hens, for winter (**a**) and summer (**b**).

Level N1 eggs in aviary 43W were approximately 2 g heavier in both winter and summer. This was also noted in the 86W aviary, with a difference in weight of approximately 6 g. The variability of egg weight values was more accentuated in 86W due to thermal conditions and bird age. In the winter, the highest-weight eggs were obtained in lines L1 and L5, and in the summer, in line L1. The difference between the egg weight values, in the lines and levels, can be spatially visualized as shown in Figure 4. This clearly shows where attention by engineering and management to improve interior thermal conditions would be most effective.

The spatial distribution of temperature, relative humidity, and egg weight in Figure 3 and Figure 4 highlights that greater egg weight values were in those regions of aviaries with temperatures within the thermal comfort zone as defined by Ferreira [8], amongst others. In winter, the air temperature inside the aviaries was between 19 and 23.5 °C, that is, within the comfort zone.

The lowest egg weight values ranged between 59 and 61 g in the 43W aviary and 62 and 66 in the 86W aviary. Akbari et al. [32] subjected laying hens aged 42 weeks to the cold stress condition (6.8 ± 3.0 °C) and found eggs weighing close to those found in the present study in the 43W aviary, where the housed birds were 43 weeks-of-age, and the thermal condition was characterized as thermoneutral. Star et al. [33] submitted 67 to 78-week old laying hens to a temperature of 20 °C and obtained eggs with an average weight of 63.1 g. This value is close to that obtained in the upper central region of the 86W aviary in winter (Figure 4a), where the air temperature was between 21 and 23 °C (Figure 3a).

In summer, the lowest egg weight values ranged between 56 and 60 g (Figure 4b) and were concentrated in the N3 level of the aviaries. At this level, temperature values between 27 and 28 °C were measured, which exceed thermal comfort (Figure 3b). Karami et al. [8] raised hens from 42 to 45 weeks in a 32 °C environment and obtained eggs with an average weight of 58 g, a value close to that found in the 43W aviary of the present study; Hu et al. [1] reported egg weights of 53–55 g for 16–32-week-old hens raised under mild heat stress and subjected to one acute heat stress event.

The area for lighter-weight eggs in the 86W aviary in summer was smaller than the area observed in the 43W aviary (Figure 4b). The hens’ heat acclimatization can explain this observation. Mashaly et al. [34] submitted 31-week-old birds to an environment with an air temperature of 35 °C for five weeks and found a decrease in egg weight only in the first week and weight maintenance thereafter. Therefore, when suffering heat stress for a longer period, older hens undergo an acclimatization process, which reduces the effect of high temperatures on egg quality compared to young birds [35]. However, even under long-term or chronic heat stress, small improvements in the thermal environment have been reported to improve egg production and quality. For example, Hu et al. [36,37] reported greater egg production, fewer cracked eggs, heavier eggs with greater breaking force, and especially shell thickness and eggshell percentage, for hens subjected to two years of heat stress but with cooled perches [38], compared to non-cooled or no perches.

In winter, the specific gravity inside the houses had a slight but significant variation between levels (*p* < 0.1). Level N1 had the highest values of specific gravity, averaging 1091 g·mL^−1^, and level N3 had the lowest values, averaging 1085 g·mL^−1^. In summer, the spatial distribution was homogeneous, and there were no significant variations between levels, lines, and sections. The mean specific gravity was 1092 g·mL^−1^ at level N1 and 1089 g·mL^−1^ at level N3.

Castro et al. [31] verified eggs with a specific gravity of 1087 g·mL^−1^ and 1090 g·mL^−1^ in 45-week-old hens subjected to thermal comfort (21 °C) or thermal stress (32 °C), respectively,. Khatibi et al. [39] studied the quality of eggs from 52, 56, and 60-week-old laying hens housed in aviaries with an average internal temperature of 27.41 ± 2.54 °C and an average relative humidity of 35 ± 5%, during a subtropical summer and verified that for 50-week-old hens, specific gravity ranged between 1079 and 1090 g·mL^−1^. For hens at 56 weeks of age, the specific gravity found was between 1084 and 1092 g·mL^−1^, and for hens at 60 weeks of age, between 1081 and 1096 g·mL^−1^. Thus, the specific gravity values obtained in winter and summer agree with the results obtained by the authors above.

The influence of winter and summer thermal conditions on the geometric characteristics of eggs was analyzed through the spatial distribution of the egg shape index values. There was a significant difference between the egg shape index values obtained between the levels in winter (*p* < 0.05) and summer (*p* < 0.01).

**Figure 5:**
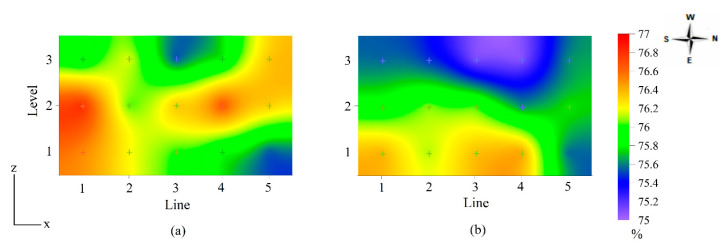
Spatial distribution, in cross-section, of the average egg shape index values obtained in the aviaries in winter (**a**) and summer (**b**).

In winter, eggs from the N2 level had higher shape index values (an average of 76.4 ± 1.0%), with eggs from the N1 and N3 levels having the lowest values (76.0 ± 1.2% and 75.9 ± 1.2%, respectively). In summer, N1-level eggs had the highest shape index values (76.2 ± 1.3%), and N3-level eggs had the lowest (75.4 ± 4.3%). Therefore, the spatial distribution of the egg shape index can be useful for depicting the difference between the average egg shape index values inside the aviaries, as shown in Figure 5.

The observed highest shape index values from the N2 level in the winter, around 76%, occurred with a mean air temperature of about 23 °C (Figure 3a and Figure 5a). By contrast, during summer, the lowest shape index values, 75%, were verified in the aviary region with air temperature values between 27 and 28 °C, outside the thermal comfort range for hens (Figure 3b and Figure 5b).

The egg shape index value found in winter was about 1% higher than that found by Orguz et al. [40] at an environmental temperature of 18 °C, but about 1% lower than that described by Akbari et al. [32] for a cold stress condition (6.8 °C). On the other hand, the summer and winter shape indexes were 2% higher than those obtained by Torki et al. [13], who subjected laying hens to thermoneutral environments (17 °C), and 4% higher than those obtained in heat stress (32 °C), which shows the influence of thermal conditions on the shape index.

The shape index is an external indicator of egg quality and is used to classify eggs based on their size. Eggs can be classified according to their shape into long, normal, and round. Long eggs have a shape index less than 72, normal eggs between 72 and 76, and round eggs greater than 76 [41]. The present study found uniformly normal oval eggs with a mean shape index between 75% and 76%.

The influence of winter and summer thermal conditions on shell quality was also analyzed through the spatial distribution of shell weight and thickness inside the aviary. For shell weight, the effect of levels (*p* < 0.001) and lines (*p* < 0.001) was significant in both winter and summer. However, only the line effect (*p* < 0.1) was significant in the summer for shell thickness. Table 6 summarizes mean eggshell weight and thickness by level (N) and line (L) factors for winter and summer. There was no significant effect (*p* > 0.05) of the level (N) and line (L) factors in winter and the level (N) factor in summer on the average value of eggshell thickness (mm), therefore, in Table 6 the means and standard deviation were followed by “ns”.

**Figure 6:**
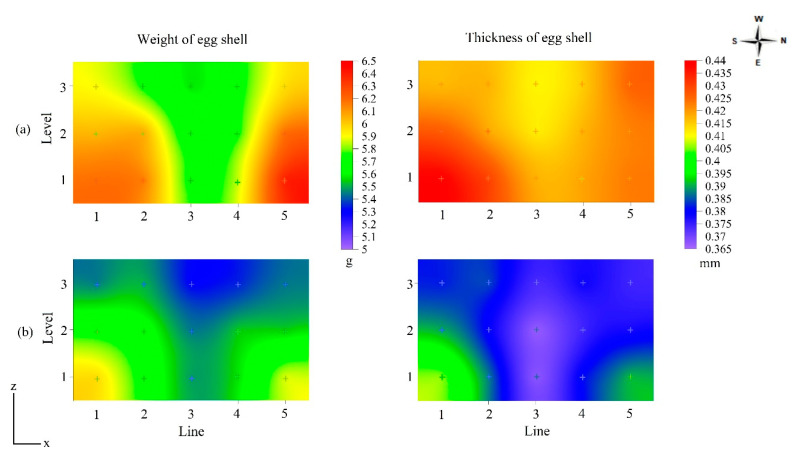
Spatial distribution, in cross-section, of the mean values of shell weight (g) and thickness (mm) in five aviaries obtained in winter (**a**) and summer (**b**).

The overall mean eggshell weight was 5.93 g and 5.57 g in summer and winter, respectively (not shown in Table 6). Level N1 and lines L1 and L5 displayed the highest values. In the summer, shell thickness values varied among lines, with the L1 line displaying the highest values. In winter, the mean value of shell thickness was 0.421 mm, and in summer, 0.381 mm. Measured variability between the mean values of shell weight and thickness inside the aviaries can be observed by a representative plot of spatial distribution in cross-section, as highlighted in Figure 6.

Across both seasons, eggshell weight varied significantly with cage level, with weights being greatest in N1 and lowest in N2 (*p* < 0.05). During the winter period, the highest shell weight values were obtained where the lowest air temperature values were measured (L1 and L5), as shown in Figure 6a.

In summer, the lowest values of shell weight and thickness were obtained in the region of the aviaries where the highest air temperature values were measured, that is, at level N3 and lines L3–L4 (Figure 6b). In these regions, the air temperature was typically between 27 and 28 °C, and the shell weight and thickness values were 5.3 g and 0.368 mm, respectively. In contrast, the spatial variation of shell thickness was not significant in winter (Table 6, *p* > 0.05). In general, greater shell thickness was observed in the region with the highest shell weight. Winter-time values of shell weight and thickness were higher than those described by Netto et al. [42], Sahin et al. [12], Torki et al. [13], and Yan et al. [43] and close to those obtained by Samiullah et al. [7] in thermoneutral environmental conditions. The measured shell thickness values corroborate those obtained by Karami et al. [11], Sahin et al. [12], and Torki et al. [13], measured in environments with elevated temperatures (32 °C and 34 °C). According to Kim et al. [44], exposure of hens to high ambient temperature results in a significant decrease in shell weight and shell thickness as these parameters are directly associated with reduced feed intake and mineral metabolism; this was also confirmed in a long-term heat stress trial by Hu et al. [1].

Regarding the effect of light intensity on the external egg quality, eggs from lines L1 and L5 (exposed to high levels of natural light, Table 3) had greater weight and greater shell thickness (Figure 4 and Figure 6); this observation contradicts that of Renema et al. [16]. In this study, within the interior lines of the aviaries (L2, L3, and L4), there was a reduction in egg weight and shell thickness with increasing light intensity from level N1 to level N3; Yildiz et al. [14], who investigated the effects of cage location and different types of lighting on egg quality parameters in a semi-confined facility with a multilevel cage system, also reported this effect.

The egg weight values in the aviary region with a light intensity of 11 lux (Table 3) were 62 g (Figure 4), a value higher than those obtained by Yuri et al. [19] and Yildirim et al. [18] when subjecting hens to an intensity of 15 lux. On the other hand, Min et al. [15] submitted laying hens to a 20-lux environment and obtained eggs with an average weight of 60.9 g, a value below that obtained in the region of the aviaries in this study with a light intensity of 20 lux, which was 64 g. These differences could be attributed to several factors, including age of laying in the other studies, breed of hen, and other uncontrolled factors.

## 4. Conclusions

The study of the spatial variability of the external quality of eggs proved to be efficient in detecting regions inside the aviary where there is a predominance of problematic eggs, including low weight, non-oval shapes, and low shell quality. These results accounted for the influence of hen age and the thermal conditions of the aviary interiors across periods of the year with mild (winter) and high (summer) temperatures; as such, a focus on those parts of the aviaries with these problematic eggs would be most beneficial for improving the overall quality of eggs from the farm.

The spatial variability of external egg quality was more pronounced in summer than in winter, regardless of hen age. Egg weight and shell quality values were lower in the upper level of cage batteries located in the center of the aviary, where cage temperatures tended to be highest.

The light intensity distribution within the aviary presented significant variability, and its effect on external egg quality was not as evident as the effect of thermal conditions. Detailed studies in this regard must be performed to obtain thelight intensity threshold that can harm egg quality in open aviaries of the sort studied, which are common in tropical and subtropical regions.

The eggs’ external quality and knowledge of their spatial distribution can be used as an economic loss indicator and an aid in nutritional and aviary thermal management. It may also be advisable to develop independent management of egg collection in these areas of the aviary if design and management improvements are found to be unhelpful.

## Figures and Tables

**Table 1:** Average daily air temperature (°C) and relative humidity (%) inside the laying hens’ aviaries in winter and summer.

Aviaries	Temperature (°C)		Relative Humidity (%)	
	Winter	Summer	Winter	Summer
Aviary 43W	21.8 ± 1.0	26.2 ± 0.5	58 ± 3.0	64 ± 1.7 b
Aviary 56W	21.9 ± 0.9	27.1 ± 0.6	57 ± 2.7	67 ± 1.5 a
Aviary 69W	21.2 ± 1.0	26.9 ± 0.5	61 ± 2.6	68 ± 1.6 a
Aviary 79W	22.2 ± 0.8	26.9 ± 0.5	56 ± 2.6	66 ± 1.6 ab
Aviary 86W	21.4 ± 0.9	26.8 ± 0.5	59 ± 2.9	68 ± 1.4 a

Means and standard deviations followed by a different lowercase letter in a column differ statistically from each other by Tukey’s test at 5% probability.

**Table 2:** Comparison between the mean daily temperature (°C) and relative humidity (%) obtained in the 43W aviary, in winter and summer, for the interaction between the level (N) and line (L) factors.

Temperature (°C)						
Winter		L1	L2	L3	L4	L5
	N1	19.6 ± 2.8 a A	21.7 ± 1.5 a A	21.3 ± 1.2 a A	19.1 ± 0.9 b A	19.2 ± 1.9 a A
	N2	20.9 ± 2.0 a A	22.3 ± 1.4 a A	22.5 ± 1.1 a A	22.3 ± 1.6 a A	20.1 ± 1.7 a A
	N3	20.9 ± 1.79 a B	21.2 ± 1.3 a AB	22.2 ± 1.3 a AB	23.8 ± 1.9 a A	19.9 ± 0.9 a B
Summer		L1	L2	L3	L4	L5
	N1	24.1 ± 1.6 b BC	25.6 ± 1.7 b A	25.2 ± 1.2 b AB	24.6 ± 1.6 b AB	23.5 ± 1.5 a C
	N2	25.9 ± 0.9 a AB	26.3 ± 1.4 b A	25.8 ± 0.8 b AB	25.1 ± 1.7 b BC	24.1 ± 1.1 a C
	N3	24.6 ± 0.7 b C	28.2 ± 1.1 a AB	27.3 ± 2.1 a B	28.7 ± 0.7 a A	23.0 ± 0.8 a D
**Relative humidity (%)**						
Winter		L1	L2	L3	L4	L5
	N1	48 ± 5.1 a B	44 ± 4.7 a BC	39 ± 5.1 a C	42 ± 5.1 a BC	56 ± 6.1 a A
	N2	43 ± 3.9 a B	33 ± 4.5 b C	35 ± 4.6 a C	37 ± 4.1 a BC	52 ± 7.1 a A
	N3	46 ± 4.9 a AB	37 ± 4.3 ab BC	34 ± 5.1 a C	38 ± 4.1 a BC	50 ± 6.7 a A
Summer		L1	L2	L3	L4	L5
	N1	65 ± 6.7 a AB	57 ± 6.1 a CB	54 ± 4.9 a C	59 ± 3.8 a BC	67 ± 3.3 a A
	N2	62 ± 6.4 ab A	53 ± 5.7 a B	50 ± 4.0 a B	60 ± 7.2 a A	61 ± 8.1 a A
	N3	59 ± 6.8 b AB	51 ± 6.5 a C	50 ± 1.9 a C	55 ± 2.6 a BC	64 ± 4.6 a A

Means and standard deviations followed by the same uppercase letters in the row and lowercase letters in the column do not differ statistically from each other by Tukey’s test at 5% probability.

**Table 3:** Mean and standard deviation of light intensity (lux) obtained in the 43W aviary in winter and summer for the N × L interaction.

N × L		L1	L2	L3	L4	L5
Winter	N1	52,630 ± 4647 a A	20 ± 1 a B	11 ± 2 a B	32 ± 3 a B	3402 ± 384 a B
	N2	49,607 ± 5389 a A	43 ± 7 a B	18 ± 4 a B	29 ± 4 a B	4341 ± 418 a B
	N3	9904 ± 878 b A	448 ± 24 a B	784 ± 55 a B	1300 ± 59 a B	5002 ± 462 a B
		L1	L2	L3	L4	L5
Summer	N1	10,626 ± 2291 a A	54 ± 4 a B	64 ± 48 a B	39 ± 25 a B	9893 ± 2535 a A
	N2	10,656 ± 2175 a A	82 ± 46 a B	74 ± 78 a B	56 ± 37 a B	10,385 ± 2326 a A
	N3	7056 ± 998 b A	170 ± 73 a B	229 ± 77 a B	880 ± 151 a B	7774 ± 1545 a A

Means and standard deviations followed by the same letter, uppercase in the row and lowercase in the column, do not differ statistically from each other by the Tukey test at 5% probability.

**Table 4:** Mean external egg quality measurements depend on the season of the year, the age of the hens, and their interactions.

Egg Weight (g)					
Season × Age (weeks)					
	43	56	69	79	86
Winter	62.4 ± 0.5 a B	62.9 ± 0.3 a B	65.6 ± 0.8 a A	66.2 ± 0.8 a A	66.9 ± 0.6 a A
Summer	59.8 ± 0.9 b B	60.9 ± 0.4 a AB	60.7 ± 0.9 b AB	61.6 ± 0.7 b AB	62.5 ± 0.7 b A
**Specific Gravity (g·mL^−1^)**					
Season	Winter	Summer			
	1.086 ± 0.008 b	1.090 ± 0.004 a			
Age (weeks)	43	56	69	79	86
	1.089 ± 0.001 ns	1.090 ± 0.003 ns	1.089 ± 0.004 ns	1.084 ± 0.008 ns	1.087 ± 0.010 ns
**Egg Shape Index (%)**					
Season	Winter	Summer			
	76.2 ± 0.5 a	75.8 ± 0.8 b			
Age (weeks)	43	56	69	79	86
	76.0 ± 0.5 ns	76.5 ± 0.4 ns	75.9 ± 0.7 ns	75.7 ± 0.5 ns	75.6 ± 0.9 ns
**Shell Weight (g)**					
Season	Winter	Summer			
	5.9 ± 0.2 a	5.6 ± 0.1 b			
Age (weeks)	43	56	69	79	86
	5.6 ± 0.2 ns	5.7 ± 0.1 ns	5.8 ± 0.3 ns	5.8 ± 0.2 ns	5.8 ± 0.3 ns
**Percentage of Shell (%)**					
Season	Winter	Summer			
	9.2 ± 0.2 ns	9.1 ± 0.2 ns			
Age (weeks)	43	56	69	79	86
	9.2 ± 0.2 ab	9.3 ± 0.1 a	9.2 ± 0.1 ab	9.1 ± 0.1 ab	8.9 ± 0.2 b
**Shell Thickness (mm)**					
Season	Winter	Summer			
	0.421 ± 0.06 a	0.381 ± 0.03 b			
Age (weeks)	43	56	69	79	86
	0.391 ± 0.04 ns	0.424 ± 0.06 ns	0.391 ± 0.04 ns	0.385 ± 0.03 ns	0.413 ± 0.07 ns

Means and standard deviations followed by the same letter, uppercase in the row and lowercase in the column, do not differ statistically from each other by the Tukey test at 5% probability. ns: not significant at 5% probability.

**Table 5:** Comparison between the mean egg weight (g) obtained in the 43W and 86W aviaries during the winter and summer seasons for the factors level (N) and line (L), as well as the N × L interaction.

Egg Weight (g)						
**Aviary 43W**						
Winter	Level (N)	N1	N2	N3		
		63.4 ± 4.8 a	61.8 ± 4.3 b	61.9 ± 4.4 ab		
	Line (L)	L1	L2	L3	L4	L5
		62.6 ± 4.0 ab	63.2 ± 4.2 a	60.6 ± 4.9b	61.8 ± 4.0 ab	63.6 ± 5.0 a
Summer	Level (N)	N1	N2	N3		
		61.8 ± 4.3 a	60.5 ± 4.6 a	57.0 ± 4.5 b		
	Line (L)	L1	L2	L3	L4	L5
		60.2 ± 5.6 ns	61.1 ± 4.3 ns	59.3 ± 4.3 ns	58.3 ± 4.7 ns	60.1 ± 5.0 ns
**Aviary 86W**						
N × L		L1	L2	L3	L4	L5
Winter	N1	65.9 ± 5.0 a A	70.0 ± 3.9 b A	68.0 ± 3.9 a A	66.9 ± 6.6 a A	69.7 ± 4.3 a A
	N2	67.1 ± 5.3 a A	66.2 ± 6.9 ab A	66.1 ± 4.6 a A	64.8 ± 2.6 a A	68.8 ± 7.6 a A
	N3	71.8 ± 7.4 a A	61.8 ± 7.4 a B	63.3 ± 3.8 a B	64.2 ± 4.7 a AB	69.0 ± 7.0 a AB
Summer	N1	65.8 ± 3.8 a A	62.1 ± 4.2 a A	62.9 ± 3.0 a A	60.8 ± 3.4 ab A	63.4 ± 5.9 a A
	N2	65.9 ± 5.5 a A	62.3 ± 3.9 a A	61.7 ± 4.7 a A	63.7 ± 3.7 a A	62.8 ± 3.3 a A
	N3	62.3 ± 5.9 a AB	64.3 ± 4.0 a A	59.7 ± 3.2 a A	56.4 ± 4.3 b B	63.3 ± 5.5 a A

Means and standard deviations followed by the same uppercase letter in the row and lowercase in the column do not differ statistically from each other by Tukey’s test at 5% probability. ns: not significant at 5% probability.

**Table 6:** Comparison between mean shell weight (g) and shell thickness (mm) obtained in the aviaries in winter and summer for the factors level (N) and line (L).

Shell Weight (g)						
Winter	Level (N)	N1	N2	N3		
		6.1 ± 0.4 a	5.9 ± 0.3 ab	5.8 ± 0.2 b		
	Line (L)	L1	L2	L3	L4	L5
		6.0 ± 0.3 a	6.0 ± 0.3 ab	5.6 ± 0.2 c	5.8 ± 0.2 bc	6.2 ± 0.3 a
Summer	Level (N)	N1	N2	N3		
		5.7 ± 0.3 a	5.6 ± 0.5 ab	5.4 ± 0.3 b		
	Line (L)	L1	L2	L3	L4	L5
		5.7 ± 0.3 a	5.6 ± 0.2 ab	5.4 ± 0.2 b	5.5 ± 0.3 ab	5.6 ± 0.3 ab
**Shell Thickness (mm)**						
Winter	Level (N)	N1	N2	N3		
		0.426 ± 0.05 ns	0.419 ± 0.05 ns	0.417 ± 0.05 ns		
	Line (L)	L1	L2	L3	L4	L5
		0.428 ± 0.06 ns	0.422 ± 0.06 ns	0.413 ± 0.05 ns	0.417 ± 0.05 ns	0.424 ± 0.04 ns
Summer	Level (N)	N1	N2	N3		
		0.387 ± 0.03 ns	0.378 ± 0.03 ns	0.378 ± 0.03 ns		
	Line (L)	L1	L2	L3	L4	L5
		0.393 ± 0.03 a	0.384 ± 0.03 ab	0.369 ± 0.02 b	0.378 ± 0.03 ab	0.381 ± 0.03 ab

Means and standard deviations by level (N) and cage line (L). Means with the same lowercase letter on a row do not differ statistically from each other by Tukey’s test at 5% probability. ns: not significant at 5% probability.

## Data Availability

The data presented in this study are available on request from the corresponding author.
